# Sodium-glucose cotransporter 2 (SGLT-2) inhibition and kidney protection: Does improvement in kidney hypoxia play a role?

**DOI:** 10.1016/j.eclinm.2021.100983

**Published:** 2021-06-28

**Authors:** Michel Burnier

**Affiliations:** a Service of Nephrology and Hypertension, University Hospital, Lausanne, Switzerland; b Hypertension Research Foundation, St-Légier, Switzerland

The concept of suppressing renal glucose reabsorption in the proximal tubule to reduce plasma glucose levels and treat patients with diabetes has been elaborated more than 30 years ago when phlorizin, a relatively unselective inhibitor of sodium-glucose cotransporter 2 (SGLT2) was investigated [1]. This therapeutic approach of diabetes has been revived in the last 10 years with the development of several selective inhibitors of SGLT2 such as dapagliflozin, canagliflozin and empagliflozin [1]. Today, the results of several large clinical trials have demonstrated that this approach provides amazing kidney protective benefits in patients with diabetic nephropathy and perhaps, also in non-diabetic nephropathies [2]. The renal protective effects of SGLT2 inhibitors go well beyond their ability to control glycaemia and several mechanisms are proposed to explain their ability to reduce kidney outcomes including decreases in blood pressure, serum uric acid and glomerular hyperfiltration [3]. Another interesting hypothesis, linked to the reduction in glomerular hyperfiltration, is that SGLT2 inhibitors correct the intrarenal mismatch between oxygen delivery and oxygen demand characteristic of diabetes and thereby improve kidney oxygenation [4].

In an article published in *EClinicalMedicine*, Jens Christian Laursen and colleagues present the results of a randomized, double-blind, placebo-controlled crossover study assessing the acute effects of a high dose of dapagliflozin on kidney tissue oxygenation and perfusion in patients with diabetes [5]. In this study, 15 patients with type 1 diabetes and albuminuria and a mean estimated glomerular filtration of 73 ml/min/m^2^ were enrolled and randomized to receive consecutively either a single dose of 50 mg dapagliflozin or a placebo with a two-week wash-out period between the two administrations. Kidney tissue oxygenation was measured using magnetic resonance imaging (BOLD-MRI) and renal perfusion was assessed using arterial spin labeling. Measurements were done at baseline and three- and six hours after drug ingestion.

A significant improvement in renal cortical oxygenation was observed after the administration of dapagliflozin when compared to placebo. However, there was no significant amelioration of medullary oxygenation. Interestingly, the observed changes in renal tissue oxygenation were not explained by an increase in oxygen delivery as no significant changes in cortical or medullary perfusion were observed after the ingestion of dapagliflozin. These results tend to support the hypothesis that SGLT2 inhibition can ameliorate kidney oxygenation in diabetes essentially through the inhibition of the diabetes-induced hyper-reabsorption of glucose and sodium cotransport in the renal proximal tubule, a high oxygen demanding transport system. They also fit with recent modeling of the effects of SGLT2 inhibition in animals with diabetes suggesting that acute and chronic SGLT2 inhibition should decrease active oxygen consumption in the cortex by about 30% due to the reduction of the hyperfiltration that lowers proximal tubule active sodium transport [6]. Proximal tubular reabsorption of the filtered sodium is recognized as the main determinant of oxygen consumption in the kidney. This was confirmed using BOLD-MRI in humans with significant variations in renal cortex oxygenation upon changes in dietary sodium intake [7]. According to the *chronic hypoxia hypothesis*, hypoxia contributes to the progression of diabetic kidney disease triggering inflammation, extracellular matrix remodeling, capillary rarefaction, and renal tissue fibrosis [4]. Of note, a low cortical oxygenation has been reported to be an independent predictor of renal function decline in patients with various causes of chronic kidney diseases, including diabetes [8].

Although the data presented by Laursen et al. [5] fit perfectly with the hypoxia hypothesis, one should remain cautious with their interpretation. First, the authors investigated patients with type 1 diabetes and whether their results can be extrapolated to patients with type 2 diabetes remains to be demonstrated. Indeed, the pathophysiology of diabetic nephropathy differs substantially in people with type 1 and type 2 diabetes [9]. Second, to test their hypothesis, authors used a single administration of a very high dose of dapagliflozin (50 mg). For the treatment of diabetes, the maximal recommended dose of dapagliflozin is 10 mg per day and pharmacological studies have demonstrated that doses of 20–50 mg provide close to maximal SGLT2 inhibition. Whether the same acute effect can be obtained with lower doses and the improved cortical oxygenation persists during a prolonged administration should be investigated. At the dose of 10 mg, empagliflozin did not affect cortical or medullary oxygenation after one month of administration in healthy subjects [10]. Nevertheless, this latter observation does not preclude that SGLT2 inhibition improves kidney oxygenation in patients with diabetes, who present multiple hyperglycemia-associated renal pathophysiological disturbances, among which a reduced cortical oxygenation. The work present by Laursen et al. is a first indication suggesting that SGLT2 inhibition indeed improves kidney oxygenation but additional human studies are needed to confirm this hypothesis.

## Contributors

MB is the sole contributor to this commentary.

## Declaration of Competing Interest

None.
